# Pneumococcal Colonization in Healthy Adult Research Participants in the Conjugate Vaccine Era, United Kingdom, 2010–2017

**DOI:** 10.1093/infdis/jiz034

**Published:** 2019-01-28

**Authors:** Hugh Adler, Elissavet Nikolaou, Katherine Gould, Jason Hinds, Andrea M Collins, Victoria Connor, Caz Hales, Helen Hill, Angela D Hyder-Wright, Seher R Zaidi, Esther L German, Jenna F Gritzfeld, Elena Mitsi, Sherin Pojar, Stephen B Gordon, Adam P Roberts, Jamie Rylance, Daniela M Ferreira

**Affiliations:** 1 Department of Clinical Sciences, Liverpool School of Tropical Medicine; 2 Department of Parasitology, Liverpool School of Tropical Medicine; 3 Royal Liverpool University Hospital; 4 Aintree University Hospital; 5 Clinical Research Network North West Coast, National Institute for Health Research, Liverpool; 6 St George’s University of London; 7 BUGS Bioscience, London Bioscience Innovation Centre, London, United Kingdom; 8 Malawi-Liverpool-Wellcome Trust Clinical Research Programme, Blantyre, Malawi

**Keywords:** Drug resistance, microbial, herd immunity, nasal washing, *Streptococcus pneumoniae*

## Abstract

Pneumococcal colonization is rarely studied in adults, except as part of family surveys. We report the outcomes of colonization screening in healthy adults (all were nonsmokers without major comorbidities or contact with children aged <5 years) who had volunteered to take part in clinical research. Using nasal wash culture, we detected colonization in 6.5% of volunteers (52 of 795). Serotype 3 was the commonest serotype (10 of 52 isolates). The majority of the remaining serotypes (35 of 52 isolates) were nonvaccine serotypes, but we also identified persistent circulation of serotypes 19A and 19F. Resistance to at least 1 of 6 antibiotics tested was found in 8 of 52 isolates.

## Teaser

We detected pneumococcal colonization in 52 of 795 subjects, using nasal wash culture. The majority of pneumococci were nonvaccine serotypes, but we identified persistent circulation of serotypes 3, 19A, and 19F and the emergence of antimicrobial resistance in the latter years of the study.

Colonization with *Streptococcus pneumoniae* is a key precursor to invasive pneumococcal disease (IPD). Colonization is more common in children than in adults, and contact with children aged <5 years is the main risk factor for adult colonization [1]. Pneumococcal conjugate vaccines (PCVs) prevent pediatric pneumococcal disease and induce herd protection against vaccine serotypes through reductions in colonization in vaccinated children and, indirectly, unvaccinated adults [2].

In the United Kingdom, PCV13 (covering serotypes 1, 3, 4, 5, 6A, 6B, 7F, 9V, 14, 18C, 19F, 19A, and 23F) was introduced to the national immunization program in April 2010, replacing PCV7. Longitudinal studies of IPD in the United Kingdom have demonstrated serotype replacement following PCV13 introduction [3]. Serotype replacement has also been demonstrated in colonization studies of children and their families: in 2015–2016, 51.1% of children aged <5 years were colonized with nonvaccine-type pneumococci, compared with 1% who were colonized with PCV13 serotypes [4]. Parental colonization rates were low, with only 2.8% of parents testing positive in 2015–2016.

Antimicrobial resistance rates among cases of pneumococcal colonization and disease vary markedly between different European countries [5, 6]. Resistance is relatively uncommon in the United Kingdom, with <10% of IPD isolates nonsusceptible to penicillin [5].

When comparing the serotypes found in IPD and those found in childhood colonization, there is some discordance. For example, serotype 8 has emerged as the commonest disease-causing serotype [3], whereas no colonization with serotype 8 was identified in the most recent childhood survey [4]. In addition, IPD studies have identified incomplete herd protection in adults against some PCV13 serotypes, particularly the highly virulent serotypes 3 and 19A, even though childhood colonization with these serotypes was exceedingly rare. This led the researchers to conclude that many disease-causing serotypes have high case-carrier ratios or perhaps only colonize for short durations [4]. It also suggests that serotype replacement in childhood colonization is an imperfect surrogate for replacement in invasive disease.

Adults without regular contact with children may be less susceptible to the ecological effects of childhood PCV programs and thus could represent a reservoir of vaccine-type pneumococci [7]. However, studies of such populations are few, typified by low sampling yield and reliance on molecular detection methods, which limit their ability to identify serotypes and assess antimicrobial susceptibility [8].

Volunteers for the experimental human pneumococcal colonization (EHPC) research program in the Liverpool School of Tropical Medicine undergo screening for pneumococcal colonization prior to participation. In this article, we use these volunteers as a surrogate for the general healthy adult population to report colonization rates, serotype distributions, and antimicrobial susceptibility profiles in healthy adults over the first 7 years of screening.

## METHODS

The rationale, methods, and inclusion/exclusion criteria for EHPC studies have been previously reported [9]. In brief, the studies are open to healthy adults aged ≥18 years, excluding those with important risk factors for pneumococcal disease, colonization, or transmission, including cigarette smoking, close contact with children aged <5 years, healthcare work or care-provider responsibilities, steroid therapy, and respiratory or immunosuppressive comorbidities. Recent antibiotic therapy (within 2 weeks) and prior pneumococcal vaccination are also exclusion criteria. The majority of study recruitment events are held in local universities.

All volunteers underwent nasal wash screening for community-acquired pneumococcal colonization at their first visit. We reviewed all screening nasal wash specimens obtained between October 2010 and March 2017. A summary of the original studies is provided in the Supplementary Materials. All studies were approved by the local National Health Service Research Ethics Committee, and all participants provided written informed consent.

Nasal washes were performed as previously described [9]. In brief, 5 mL of 0.9% sodium chloride solution was introduced by a syringe into the nose, where it was held for a few seconds before being expelled into a sterile container. The participants were advised to occlude their pharynx (eg, by pressing their tongue against their hard palate) during the procedure, which was repeated twice in each nostril, resulting in the use of 20 mL of saline. Nasal wash samples were transported to the laboratory within 1 hour of collection, where they were plated on gentamicin/blood agar and incubated overnight at 37°C with 5% CO_2_. Community-acquired pneumococcal colonization was defined as the identification of *S. pneumoniae* by using standard microbiological techniques, with serogroup identified by the latex agglutination test. Where required, the serotype was confirmed using the Senti-SP v1.6 molecular serotyping microarray (BUGS Bioscience) as previously described [10]. We tested all isolates for susceptibility to penicillin, clarithromycin, doxycycline, levofloxacin, trimethoprim-sulfamethoxazole, and vancomycin, using disk diffusion and the Etest (bioMérieux, Basingstoke, United Kingdom), following the recommendations and clinical breakpoints of the European Committee on Antimicrobial Susceptibility Testing (EUCAST, version 7.1 [11]; Supplementary Materials). Zones of inhibition were measured by 2 independent reviewers, and all resistant isolates were tested a second time, for confirmation. We used meningitis breakpoints when interpreting penicillin minimum inhibitory concentrations.

For purposes of analysis, we assumed that maximal vaccine coverage of children aged <5 years with the primary series of PCV13 was achieved 5 years after its introduction [3]. We compared the proportion of individuals who were colonized, the proportion who were colonized with PCV13 serotypes, and the proportion of isolates displaying antibiotic-resistant phenotypes before and after 1 April 2015 (the early and late periods, respectively), by χ^2^ analysis or the Fisher exact test where appropriate, using SPSS version 24 (IBM, New York, NY).

## RESULTS

A total of 795 healthy volunteers met the inclusion criteria and underwent nasal wash screening (Table 1). The median age was 21 years (interquartile range, 20–23 years), and 452 (57%) were female. Pneumococcal colonization was detected in 52 participants (6.5%; 95% confidence interval [CI], 5.0%–8.5%).

**Table 1.** Demographic Characteristics of Participants

	Non-colonized (n = 743)	Colonized (n = 52)	Total
Age, y, median (IQR)	21 (20–23)	21 (19–23)	21 (20–23)
Female sex, no. (%)	428 (57.6)	24 (46.2)	452 (56.9)
Year of screen, no. (% of annual total)			
2010	8 (100)	0	8
2011	65 (97)	2 (3)	67
2012	129 (89.6)	15 (10.4)	144
2013	31 (96.9)	1 (3.1)	32
2014	66 (93)	5 (7)	71
2015	154 (94.5)	9 (5.5)	163
2016	178 (93.2)	13 (6.8)	191
2017	112 (94.1)	7 (5.9)	119

Abbreviation: IQR, interquartile range.

**Figure 1.**
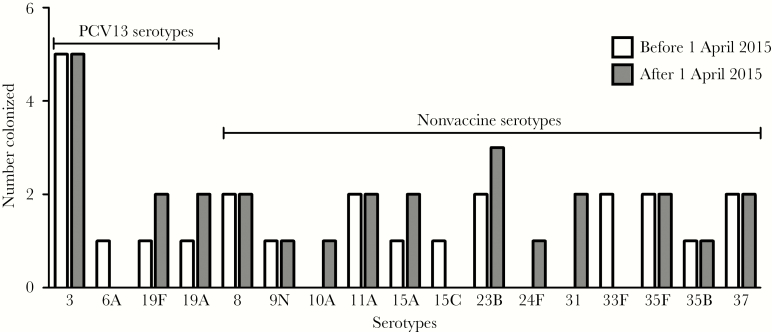
Serotype distribution among subjects colonized with vaccine serotypes (*left*) or nonvaccine serotypes (*right*) before (white bars) and after (gray bars) the 5-year anniversary of the introduction of the 13-valent pneumococcal vaccine (PCV13) in the United Kingdom.

We identified PCV13 serotypes in 17 of 52 (32.7%; Figure 1 and Supplementary Table 3). Serotype 3 was the commonest isolate (10 of 52 isolates); the next most common vaccine types were 19A and 19F (3 isolates each). The most common nonvaccine types were 23B (5 isolates) and 8, 11A, 35F, and 37 (4 isolates each). Colonization rates were not significantly different between the early and late periods (24 of 368 [6.5%] and 28 of 427 [6.6%], respectively; *P* = .98). Among colonized participants, 8 of 24 (33.3%) were carrying PCV13 serotypes before 1 April 2015, compared with 9 of 28 (32.1%) carrying PCV13 serotypes afterward (*P* = .93).

We identified resistance to at least 1 antibiotic in 8 of 52 isolates (15.4%; 95% CI, 8.0%–27.5%; Supplementary Figure 1 and Supplementary Table 4). The majority of resistant isolates (6 of 8) were nonvaccine types. The highest rate of resistance was against penicillin (5 of 8 isolates; all were susceptible to amoxicillin and ceftriaxone), followed by clarithromycin and doxycycline (4 of 8 each). We did not detect any levofloxacin or vancomycin resistance. We identified resistance to 3 antibiotic classes in 2 isolates and resistance to 2 classes in a further 4 isolates. All resistant isolates were detected after 1 April 2015 (8 of 28 [28.6%]), compared with 0 of 24 before this cutoff (*P* = .005).

## DISCUSSION

We found a higher rate of colonization than has previously been reported in the general European adult population [1, 12]. This finding is notable given that the exclusion criteria for EHPC studies resulted in all participants lacking major risk factors for pneumococcal acquisition [9]. When our results are compared to those from a recent colonization study of United Kingdom children aged <5 years and their parents, we identified more frequent colonization and a greater range of serotypes than were found in the parents but less colonization and fewer serotypes than were found in the children [4].

Although our data collection spanned the first 7 years of PCV13 implementation, the relative distribution of serotypes did not change over this time. Our colonization patterns were dominated by nonvaccine types, reflecting current trends in IPD and colonization in the United Kingdom [3, 4]. Serotype 8 is currently the commonest cause of IPD in the United Kingdom and was the joint third-most frequently detected serotype in our study, even though it was not detected in the most recent United Kingdom pediatric colonization survey [3, 4]. A cross-sectional study in 2010–2011 identified PCV13 serotypes (specifically 3, 19F, and 19A) in 5 of 36 colonized United Kingdom adults [6]; we identified ongoing circulation of these same serotypes throughout the PCV13 era. This could explain why serotypes 3 and 19A remain common in IPD even though they are rarely identified in pediatric colonization [3, 4]. This reservoir in young adults could have the potential to recolonize the pediatric population after the United Kingdom reduces the childhood PCV13 schedule from 3 to 2 doses [13]. Conversely, the failure of the current pediatric vaccine schedule to generate herd protection against these serotypes could support a call for vaccination of at-risk adults, although the 23-valent pneumococcal polysaccharide vaccine is already used for direct protection of this population in the United Kingdom.

While our study outperformed the pediatric colonization survey in detecting certain serotypes with invasive potential (eg, 3, 8, and 19A), the pediatric survey frequently identified carriage with invasive serotypes (eg, 15B/C and 10A) that were poorly represented in our cohort [4]. Therefore, it seems that colonization surveys should optimally include a wider range of subjects, rather than being restricted to households with children. This would maximize their potential to quantify serotype replacement and complement IPD surveillance in guiding future vaccine formulation recommendations.

Colonization studies have typically focused on children and their close contacts, to maximize the yield from screening. We are aware of one other study that deliberately recruited a control cohort of adults who lacked close contact with children [8]. The authors detected colonization in 10% of Dutch parents (29 of 298) versus 2% of controls (5 of 323), using culture-based methods. Molecular testing increased the yield to 7% in controls (21 of 323), similar to our findings, which were based on culture-based methods in United Kingdom adults.

Antimicrobial resistance in pneumococci is a growing global health concern [14]. We detected the emergence of antimicrobial resistance during the later period of this study, with no resistant isolates identified during the early period. However, a cross-sectional study including data from United Kingdom primary care practices in 2010 identified cases of resistant pneumococcal colonization in adults, suggesting that the absence of resistance in the early years of our data collection may be due to either chance or specific local factors [1]. The resistance profiles of different pneumococcal serotypes in adult colonization have not been previously reported in the United Kingdom. Although our numbers are small, it is concerning that the majority of resistant isolates displayed resistance to >1 class of antibiotic. Clustering of resistance in nonvaccine types suggests that the childhood PCV13 program is unlikely to reduce this reservoir of antimicrobial resistance in the community.

The main constraint of the study is the limited demographic and medical history data available. However, the strict inclusion criteria mandated by EHPC studies should have resulted in a homogeneous study population, defined by the absence of significant risk factors for pneumococcal colonization. Another limitation is that our sampling was performed in a single city. Culture-based colonization determination, allowing accurate serotyping and resistance measurement, is a strength of our study—molecular testing of oropharyngeal samples may have increased our yield but would not have allowed phenotypic confirmation of antimicrobial resistance [8]. Our use of nasal wash specimens, rather than nasal or nasopharyngeal swab specimens, may have contributed to the high pneumococcal yield obtained in this study [15]: a study using nasal swabs in >3000 United Kingdom adults only identified colonization in 1.8% of participants [1].

In summary, we described pneumococcal colonization in a population of adults not normally targeted by colonization studies, including the persistence of some PCV13 serotypes, epidemiologically important nonvaccine types, and emerging antimicrobial resistance. Inclusion of a wider sample of adults in colonization studies would complement ongoing surveillance of IPD isolates to inform national pneumococcal vaccination policies.

## Supplementary Data

Supplementary materials are available at The *Journal of Infectious Diseases* online. Consisting of data provided by the authors to benefit the reader, the posted materials are not copyedited and are the sole responsibility of the authors, so questions or comments should be addressed to the corresponding author.

## Supplementary Material

jiz034_suppl_Supplementary_Appendix

## Notes

***Acknowledgments.*** We thank all volunteers; the current and past members of the Experimental Human Pneumococcal Carriage team, including statisticians, administration, clinical, and laboratory staff; the staff of the Royal Liverpool University Hospital Clinical Research Unit; Nick Feasey and Neil French, for helpful advice; the members of the Data Safety and Monitoring Committee (Brian Faragher, David Lalloo, and Robert Read); and the research governance staff of the Liverpool School of Tropical Medicine, and the Royal Liverpool and Broadgreen University Hospitals National Health Service Trust, for their support.

***Disclaimer.*** The funders had no role in study design, data analysis, manuscript preparation or decision to submit to publication.

***Financial support.*** This work was supported by the Jean Clayton Fund at the Liverpool School of Tropical Medicine (to H. A. and E. N.). The Experimental Human Pneumococcal Carriage program is funded by the Bill and Melinda Gates Foundation and the United Kingdom Medical Research Council, supported by the National Institute for Health Research Clinical Research Network (North West Coast)..

***Potential conflicts of interest.*** A. R. reports lecture fees from Merck Sharp and Dohme, outside the submitted work. J. H. reports grants from Pfizer, Sanofi Pasteur, and GSK, outside the submitted work, and being a cofounder, board member, and shareholder of BUGS Bioscience, a not-for-profit spin-out company of St George’s University of London, from which he receives no income. K. G. reports being subcontracted to BUGS Bioscience, from which she receives no income. All other authors report no potential conflicts.
