# Supplementary material for: Pneumococcal Colonization in Healthy Adult Research Participants in the Conjugate Vaccine Era, United Kingdom, 2010–2017
Source: J Infect Dis. 2019 Jan 28;219(12):1989–93. doi: 10.1093/infdis/jiz034 (PMC6534187; doi:10.1093/infdis/jiz034)
Supplement: jiz034_suppl_Supplementary_Appendix [file jiz034_suppl_supplementary_appendix.docx]

**Supplementary appendix**

**Table S1: Original experimental human pneumococcal colonization (EHPC) studies**

| **Study title/description** | **Year commenced** | **Number screened** | **Research Ethics Committee reference number** | **Other reference numbers** | **Notes** |
| --- | --- | --- | --- | --- | --- |
| Dose ranging and reproducibility | 2010 | 217 | 11/NW/0592 | - | Some results published in [1] |
| A study to evaluate the effect of pneumococcal conjugate vaccination on experimental human pneumococcal colonization | 2013 | 100 | 12/NW/0873 | EudraCT 2012-005141-20 | Published in [2]  All baseline nasal washes were taken before the administration of any vaccine. |
| A study to evaluate the effect of Gen-004 vaccine (Genocea Biosciences) on experimental human pneumococcal colonization | 2014 | 96 | 14/NW/0355 | NCT02116998 | In follow-up, preliminary results presented [3].  This was a Phase 2 study of an experimental, serotype-independent vaccine, and the first nasal washes were taken 64 days post-vaccine. However, the preliminary results suggest that this vaccine does not have a statistically significant effect on colonization. If this vaccine does in fact affect colonization, our results would be biased towards underestimating true natural colonization rates. There is no reason to believe that serotype distributions or rates of antimicrobial resistance were biased by including participants from this trial. |
| Pilot study of repeated mucosal sampling on experimental human pneumococcal colonization | 2015 | 21 | 15/NW/0146 | - | Published in [4] |
| A study of the effect of Live Attenuated Influenza Vaccine on experimental human pneumococcal colonization | 2015 | 345 | 14/NW/1460 | EudraCT 2014-004634-26 | Publication pending.  All baseline nasal washes were taken before the administration of any vaccine. |
| The effect of age on immune function | 2016 | 16 | 16/NW/0031 | ISRCTN10948363 | In follow-up; the dataset for the community-acquired colonization study was finalised before this study completed recruitment.  This was the only study to enrol participants aged ≥50 years. Pneumococcal polysaccharide vaccination was not an exclusion criterion for this study, as it is not considered to affect colonization [5]; two of the participants had received this vaccine. |

**Nasal wash methodology**

We used a modified Naclerio method in all EHPC studies [6, 7]. This has been validated by our team as being at least as sensitive as, and better tolerated than the World Health Organisation gold standard nasopharyngeal swab for the detection of pneumococci in adults [8, 9]).

In the Naclerio method, 5mL of 0.9% sodium chloride solution (normal saline) is introduced using a syringe and held for a few seconds in the nose before being expelled in to a sterile container. The participant is advised to occlude their pharynx, either by pressing their tongue against their hard palate, or by “holding a swallow mid-way through”. The procedure is repeated twice in each nostril, thus using 20mL saline in total. In the event of nasal wash loss (e.g. through inadvertent swallowing) the procedure may then be repeated to obtain an adequate specimen (defined as ≥10mL saline recaptured) using up to an additional 10mL of saline. Nasal wash samples were kept at ambient temperature and transported to the laboratory within one hour of collection.

**Bacterial culture**

Nasal wash samples were centrifuged for ten minutes at 3,345g, and the supernatant separated from the residual pellet. The pellet was resuspended in 100µL STGG, 20µL of which was streaked on a gentamicin/blood agar plate (Columbia agar with 5% horse blood, ThermoFisher, Basingstoke, UK) and incubated overnight at 37°C in 5% carbon dioxide. (The gentamicin suppresses competing respiratory pathogens, thus maximising the chance of pneumococcal detection.) Pneumococci were defined using classical microbiological criteria:

- 1. Typical draughtsman-like colony morphology on agar;
  2. The presence of α-hemolysis;
  3. Optochin sensitivity;
  4. Solubility in bile salts;
  5. Gram-positive diplococci on microscopy.

The serogroup was identified using a commercially available latex agglutination test (PneumoLatex, Statens Serum Institut, Copenhagen, Denmark) and isolates were stored on glass beads at -80°C. Molecular serotyping was performed on genomic DNA using the Senti-SP v1.6 molecular serotyping microarray (BUGS Bioscience)—see published work for methodology [10, 11].

**Antimicrobial susceptibility testing**

We screened for antimicrobial resistance using disc diffusion according to methodology recommended by the European Committee on Antimicrobial Susceptibility Testing (EUCAST, version 7.1 [12]). Antimicrobial discs and plates (Mueller-Hinton agar supplemented with 5% defibrinated horse blood and 20 mg/L β-nicotinamide adenine dinucleotide) were obtained from ThermoFisher (Basingstoke, UK). If minimum inhibitory concentration (MIC) confirmation was required, this was done by gradient diffusion (Etest, BioMérieux, Basingstoke, UK) according to the manufacturer’s instructions. *Streptococcus pneumoniae* ATCC 49619 was used as a quality control strain. The agents chosen, concentrations within each disc, susceptibility breakpoint diameters and MIC breakpoints are shown in **Table S2**.

**Table S2: EUCAST disc diameter and MIC breakpoints**

| **Specific agent used for AMR screening by disc diffusion** | **Concentration of agent within disc** | **EUCAST zone diameter breakpoint (mm)** | | **Agents for which MICs were measured (if resistance identified by disc diffusion)** | **MIC breakpoint (mg/L)** | |
| --- | --- | --- | --- | --- | --- | --- |
|  |  | **S ≥** | **R <** |  | **S ≤** | **R >** |
| Oxacillin | 1µg | 20 | - | Benzylpenicillin (meningitis) | 0.06 | 0.06 |
|  |  |  |  | Amoxicillin | 0.5 | 2 |
|  |  |  |  | Ceftriaxone | 0.5 | 0.5 |
| Erythromycin | 15µg | 22 | 19 | Clarithromycin | 0.25 | 0.5 |
| Norfloxacin | 10µg | 11 | - | Levofloxacin | 2 | 2 |
| Tetracycline | 30µg | 25 | 22 | Doxycycline | 1 | 2 |
| Trimethoprim-sulfamethoxazole | 1.25/23.75µg | 18 | 15 | *Not required* | *Not required* | *Not required* |
| Vancomycin | 5µg | 16 | 16 | *Not required* | *Not required* | *Not required* |

**Table S3: Number of volunteers colonized with each serotype, before and after the five-year anniversary of PCV13 introduction**

| **Serotype** | **Pre 01/04/2015** | **Post 01/04/2015** | **Total** |
| --- | --- | --- | --- |
| **3^*†^** | 5 | 5 | 10 |
| **23B** | 2 | 3 | 5 |
| **8^†^** | 2 | 2 | 4 |
| **11A^†^** | 2 | 2 | 4 |
| **35F** | 2 | 2 | 4 |
| **37** | 2 | 2 | 4 |
| **15A** | 1 | 2 | 3 |
| **19F^*†^** | 1 | 2 | 3 |
| **19A^*†^** | 1 | 2 | 3 |
| **9N^†^** | 1 | 1 | 2 |
| **31** | 0 | 2 | 2 |
| **33F^†^** | 2 | 0 | 2 |
| **35B** | 1 | 1 | 2 |
| **6A^*^** | 1 | 0 | 1 |
| **10A^†^** | 0 | 1 | 1 |
| **15C** | 1 | 0 | 1 |
| **24F** | 0 | 1 | 1 |
| **Total** | **24** | **28** | **52** |

* included in PVC13; † included in PPV23

Of note, the molecular microarray suggested dual colonization in one subject—99% of the signal was attributed to serotype 37 and 1% to 19F; we classified the participant as colonized with serotype 37.

**Table S4: Antimicrobial susceptibility profiles among the eight isolates with any resistance detected**

| **Date isolated** | **Age (yrs)** | **Sex** | **Serotype** | **Susceptibility to antimicrobial agents** | | | |
| --- | --- | --- | --- | --- | --- | --- | --- |
|  |  |  |  | *Penicillin G* | *Clarithromycin* | *Doxycycline* | *TMP-SMX* |
| Apr 07 2015 | 27 | F | 24F | S | S | S | **R** |
| Apr 21 2015 | 23 | M | 15A | **R** | **R** | **R** | S |
| Oct 29 2015 | 18 | M | 15A | **R** | **R** | S | S |
| Feb 18 2016 | 21 | F | 23B | **R** | S | S | **R** |
| Jul 06 2016 | 57 | M | 23B | **R** | S | S | **R** |
| Oct 13 2016 | 20 | M | 3* | S | S | **R** | S |
| Nov 16 2016 | 19 | F | 8 | S | **R** | **R** | S |
| Feb 08 2017 | 20 | M | 19F* | **R** | **R** | **R** | S |

* PCV13 serotype; R: Resistant; S: Sensitive; TMP-SMX: Trimethoprim-sulfamethoxazole.


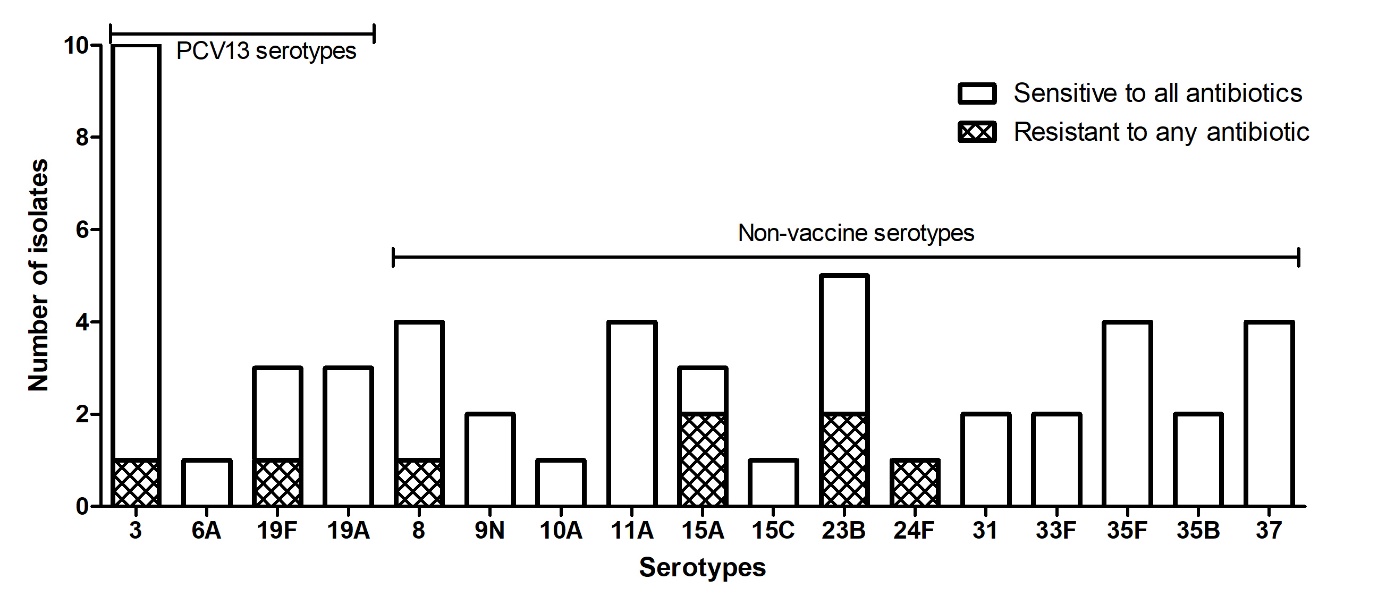


**Figure S1:** Numbers of isolates from each serotype in whom resistance was identified to any of six antimicrobials tested

Solid components represent pan-sensitive isolates, while cross-hatched components denote numbers resistant to any antibiotic tested.

**References**

1. Ferreira DM, Neill DR, Bangert M, et al. Controlled human infection and rechallenge with Streptococcus pneumoniae reveals the protective efficacy of carriage in healthy adults. Am J Respir Crit Care Med **2013**; 187:855-64.

2. Collins AM, Wright AD, Mitsi E, et al. First Human Challenge Testing of a Pneumococcal Vaccine. Double-Blind Randomized Controlled Trial. American Journal of Respiratory and Critical Care Medicine **2015**; 192:853-8.

3. Gordon SB. GEN-004 Vaccine is safe, immunogenic and reduces acquisition of colonization in experimental human pneumococcal challenge model. The International Symp​o​sium​​ on Pneumococci and Pneumococc​al Diseases. Glasgow, **2016**.

4. Jochems SP, Piddock K, Rylance J, et al. Novel Analysis of Immune Cells from Nasal Microbiopsy Demonstrates Reliable, Reproducible Data for Immune Populations, and Superior Cytokine Detection Compared to Nasal Wash. PloS one **2017**; 12:e0169805.

5. Moberley S, Holden J, Tatham DP, Andrews RM. Vaccines for preventing pneumococcal infection in adults. The Cochrane database of systematic reviews **2013**:Cd000422.

6. Naclerio RM, Meier HL, Kagey-Sobotka A, et al. Mediator release after nasal airway challenge with allergen. Am Rev Respir Dis **1983**; 128:597-602.

7. Gritzfeld JF, Wright AD, Collins AM, et al. Experimental human pneumococcal carriage. Journal of visualized experiments : JoVE **2013**.

8. Gritzfeld JF, Roberts P, Roche L, El Batrawy S, Gordon SB. Comparison between nasopharyngeal swab and nasal wash, using culture and PCR, in the detection of potential respiratory pathogens. BMC research notes **2011**; 4:122.

9. Satzke C, Turner P, Virolainen-Julkunen A, et al. Standard method for detecting upper respiratory carriage of Streptococcus pneumoniae: updated recommendations from the World Health Organization Pneumococcal Carriage Working Group. Vaccine **2013**; 32:165-79.

10. Satzke C, Dunne EM, Porter BD, Klugman KP, Mulholland EK. The PneuCarriage Project: A Multi-Centre Comparative Study to Identify the Best Serotyping Methods for Examining Pneumococcal Carriage in Vaccine Evaluation Studies. PLoS medicine **2015**; 12:e1001903.

11. Newton R, Hinds J, Wernisch L. Empirical Bayesian models for analysing molecular serotyping microarrays. BMC bioinformatics **2011**; 12:88.

12. EUCAST. The European Committee on Antimicrobial Susceptibility Testing. Breakpoint tables for interpretation of MICs and zone diameters. Version 7.1. <http://www.eucast.org>, **2017**.
